# Biodegradation of ramie stalk by *Flammulina velutipes*: mushroom production and substrate utilization

**DOI:** 10.1186/s13568-017-0480-4

**Published:** 2017-09-12

**Authors:** Chunliang Xie, Wenbing Gong, Li Yan, Zuohua Zhu, Zhenxiu Hu, Yuande Peng

**Affiliations:** Institute of Bast Fiber Crops, Chinese Academy of Agricultural Sciences, Changsha, 410205 People’s Republic of China

**Keywords:** *Flammulina velutipes*, Lignocellulolytic enzymes, Lignocellulolytic degradation, Ramie stalk

## Abstract

In the textile industry, ramie stalk is byproducts with a low economic value. The potential use of this leftover as a substrate ingredient for *Flammulina velutipes (F. velutipe)* cultivation was evaluated. The degradation and utilization of ramie stalk by *F. velutipes* was evaluated through mushroom production, lignocelluloses degradation and lignocellulolytic enzymes activity. The best substrate mixture for *F. velutipes* cultivation comprised 50% ramie stalk, 20% cottonseed hulls, 25% wheat bran, 4% cornstarch and 2% CaCO_3_. The highest biological efficiency of fruiting bodies was reached 119.7%. *F. velutipes* appears to degrade 12.7–32.0% lignin, 14.4–30.2% cellulose and 9.3–25.7% hemicellulose during cultivation on the different substrates. The results of enzymes activities showed that laccase and peroxidase were higher before fruiting; while cellulase and hemicellulase showed higher activities after fruiting. The biological efficiency of fruiting bodies was positively correlated with the activities of cellulase, hemicellulase and ligninolytic enzyme. The results of this study demonstrate that ramie stalk can be used as an effective supplement for increasing mushroom yield in *F. velutipes.*

## Introduction

Ramie, known as China grass (*Boehmeria nivea* L. *Gaudich*., *Urticaceae*), is an industrially important crop which is cultivated in China, Brazil, South Korea, Lao PDR, Philippines, India, and Thailand (Zhu et al. 2014). China is the world’s largest producer of ramie. In China, more than 1,000,000 ton of ramie residue such as ramie stalk is produced as textile industry byproduct in 2013 year which have very little or no economic value (Zhou et al. 2015). The ramie stalk is predominately composed of cellulose, hemicellulose and lignin. Many edible white rot fungi can utilize a variety of lignocellulosic residues by producing several extracellular secreted enzymes including cellulases, hemicellulases, pectinase and ligninase (Isikhuemhen et al. 2012; Levin et al. 2012; Varnai et al. 2014; Wang et al. 2013). So cultivation of mushrooms on ramie byproducts may be one of the possible solutions to converting these agro-wastes into accepted edible biomass of high and useful market value.

*Flammulina velutipes* (*F. velutipes*) was also called as golden needle mushroom or winter mushroom. Fruiting bodies of *F. velutipes* possess delicious taste and medicine use for their rich nutrition (Jing et al. 2014; Kang et al. 2014). It has also been highly valued as a functional food for its good antioxidant, anti-inflammatory, immunomodulatory, anti-tumour, and cholesterol-lowering effects (Chen et al. 2015; Wu et al. 2014; Xia 2015; Yan et al. 2014). The production and marketing potential of *F. velutipes* in China and the world is promising. Over 300,000 tons of this mushroom are produced every year (Park et al. 2014). Many private entrepreneurs are interested in its commercial cultivation (Tsai et al. 2017). In present, *F. velutipes* has been cultivated on several lignocellulosic substrates including cotton seed shells, sawdust, sugarcane bagasse and corn cobs (Huang et al. 2015; Jing et al. 2014). Demand for sawdust and cotton seed hull is increasing following the large number of poultry industry and mushroom cultivation, thus making it difficult and expensive for commercial mushroom growers to get sawdust and cotton seed hull. Within this context, growers tend to select the best and the least expensive, locally available substrate materials. Ramie stalks are easy to get in China and other countries.

The objective of this study was to investigate the possibility of using ramie stalks either as a complete substrate, or as a supplement of wheat straw and cotton seed hull based substrates in *F. velutipes* cultivation. Some characteristics of substrates prepared by ramie stalks alone, and its mixtures with wheat straw, cotton seed hull in different ratios were compared, including their effects on spawn run time, yield and biological efficiency. The degradation and utilization ability of *F. velutipes* to ramie stalks substrate were evaluated comprehensively from the content changes of cellulose, hemicellulose and lignin in medium. The relationships between fruit body production and ligninolytic enzyme activities were also determined.

## Materials and methods

### Substrate preparation and inoculation

The *F. velutipes* (CGMCC5.786) was obtained from the China general microbiological culture collection center. Raw materials including cottonseed hulls, wheat bran and cornstarch were obtained from local grocery stores. Ramie stalk was obtained from the Institute of Bast Fiber Crops, Chinese Academy of Agricultural Sciences. The ramie stalk was chopped to 2–5 mm pieces before use. The different combinations of ramie stalk, cottonseed hulls, wheat bran and cornstarch used as cultivation substrates are shown in Table 1. Each treatment contained 300 g dry substrate. Each substrate combination was mixed and moisture content adjusted to 70% before use. The substrate-supplement mixtures were filled in 33 × 17 cm^2^ polypropylene bags, tightly packed, then securely closed with plastic ties, and sterilised at 121 °C for 3 h. After cooling, each bag was spawned with 10% (w/w) mushroom mycelia grown on cottonseed hulls on a dry weight basis of substrate. The temperature, relative humidity and light were maintained at 18–24 °C, 60–70% and dark, respectively.

**Table 1:** Composition of the substrates used for *Flammulina velutipes* cultivation

Substrate number	Ramie stalk (%)	Cottonseed hulls (%)	Wheat bran (%)	Cornstarch (%)	CaCO_3_ (%)	Carbon/nitrogen ratio
1	10	60	25	4	1	40/1
2	20	50	25	4	1	38/1
3	30	40	25	4	1	43/1
4	40	30	25	4	1	38/1
5	50	20	25	4	1	31/1
6	60	10	25	4	1	28/1
7	70	0	25	4	1	25/1
8	0	70	25	4	1	43/1
9	98	0	0	0	2	78/1

### Mycelium growth measurements

After the incubation period had ended, mycelium growth on substrates was recorded. The radial growth of the mycelium was estimated from the fastest and slowest mycelium growth front point. 20 replicates were averaged.

### Mushroom cultivation

Upon full colonization, the bags were transferred to the mushroom cultivation room with 85–90% relative humidity, 12–15 °C and 12 h light cycle. The mushrooms were harvested before caps started to invert. Fruit bodies in each bag were manually harvested and weighed. After two flushes, the total mushroom yield was calculated. Biological efficiency (BE) was defined and calculated for each substrate as following: weight of fresh fruiting bodies divided by initial weight of dry substrate multiplied by 100. 20 replicates were conducted and the average BE for each substrate was determined.

### Cellulose, hemicelluloses and lignin contents determination

The contents of cellulose, hemicelluloses and lignin in ramie stalk medium were estimated and the changes occurring during fructification were calculated. The cellulose, hemicellulose and lignin contents of pre- and post-treatment were determined by method as described (Garcia-Maraver et al. 2013; Pasangulapati et al. 2012). Total lignin content was determined by two-step acid hydrolysis method according to laboratory analytical procedure of the national renewable energy laboratory (Studer et al. 2011). The experiment was conducted three times and the average value for each substrate was determined.

### Enzyme activity determination

The enzymes were extracted from 10 g of different substrates before and after fruiting using 100 mL of the extraction in 0.1 M sodium phosphate buffer (pH 6.5). The enzyme activity results of experiments were performed 20 replicates and the average enzyme activity for each substrate was determined. Laccase activity was determined according to the method described by Aracri et al. (2011). The peroxidase activity was assayed according to the method as described (Coconi-Linares et al. 2014). Carboxymethyl cellulase (CMCase), 1,4-β-exoglucanase and 1,4-β-glucosidase were determined according to the method described by Kaufman et al. (Adlakha et al. 2012; Gomaa 2013; Nakatani et al. 2010). 1,4-β-xylosidase and xylanase was determined as described (Gupta et al. 2011; Vetrovsky et al. 2013).

### Statistical analysis

One-way analysis of variance (ANOVA) was used to test the equality of treatment means in each group. Multiple comparison t tests (Fisher’s Protected LSD) conducted within each group to compare each treatment mean when the overall F-ratio was found to be statistically significant (α = 0.05, P < 0.05) for both groups. Statistical analyses of data were conducted using SPSS (v21). Correlation coefficients (R) between biological efficiency and lignocellulose degradation, enzyme activities were computed.

## Results

### Mycelium growth and biological efficiency

The results in Table 2 showed that the highest mycelium growth was measured on the substrate containing 50% ramie stalk, 20% cottonseed hulls, 25% wheat bran, 4% cornstarch and 2% CaCO_3_. Mycelial colonization on all the mediums excluding substrate number 9 was 37 ± 1 days, and 98% proportions of ramie stalk substrate was 45 days. It was noticed that addition of ramie stalk in proportions ranging 10–50% increased mycelium expansion. After that, increasing the proportion of ramie stalk resulted in inhibition of mycelium growth. The highest mycelium growth density was appeared in 50% proportions of ramie stalk medium, but the density of the mycelium was comparatively poor on 70% and 98% ramie stalk substrate.

**Table 2:** Means and standard deviation for surface mycelia density, spawn run time, yield and biological efficiency for first and second break production of *Flammulina velutipes* influenced by different substrates

Substrate number	Surface density	Mycelia spawn run time (day)	Total fruit body yield (g)	Biological efficiency (%)
1	+++	38	306.7 ± 16.2	102.2 ± 13.8
2	+++	37	315.2 ± 22.5	105.0 ± 11.7
3	+++	37	327.8 ± 19.3	109.3 ± 22.8
4	+++	37	332.5 ± 23.4	111.1 ± 25.7
5	+++	36	359.1 ± 15.3^a^	119.7 ± 13.5^a^
6	+++	37	337.3 ± 16.71	112.4 ± 17.8
7	++	37	298.4 ± 22.8	99.5 ± 19.4
8	+++	38	340.5 ± 11.4	113.5 ± 22.4
9	+	45	159.4 ± 13.7	53.1 ± 10.3

^a^Significant at 0.01 level

During 80 days of cultivation, two flush of the mushroom were harvested. Yield and BE of mushroom production varied in different substrates. The highest mushroom yield (359 g/300 dry substrate) and BE (119%) were obtained from the 50% proportions of ramie stalk medium; however, yield and BE were not significantly different from the 40% proportions of ramie stalk medium or from 60% proportions of ramie stalk medium. 70% proportions of ramie stalk in cultivation substrates of *F. velutipes* resulted in low mushroom production. The lowest yield was observed in the 98% proportions of ramie stalk medium. The results suggested that inclusion of ramie stalk is clearly advantageous for production of *F. velutipes* in proportions ranging 10–50%, especially in 50% ramie stalk combination.

### Lignin, cellulose, and hemicellulose degradation

**Fig. 1:**
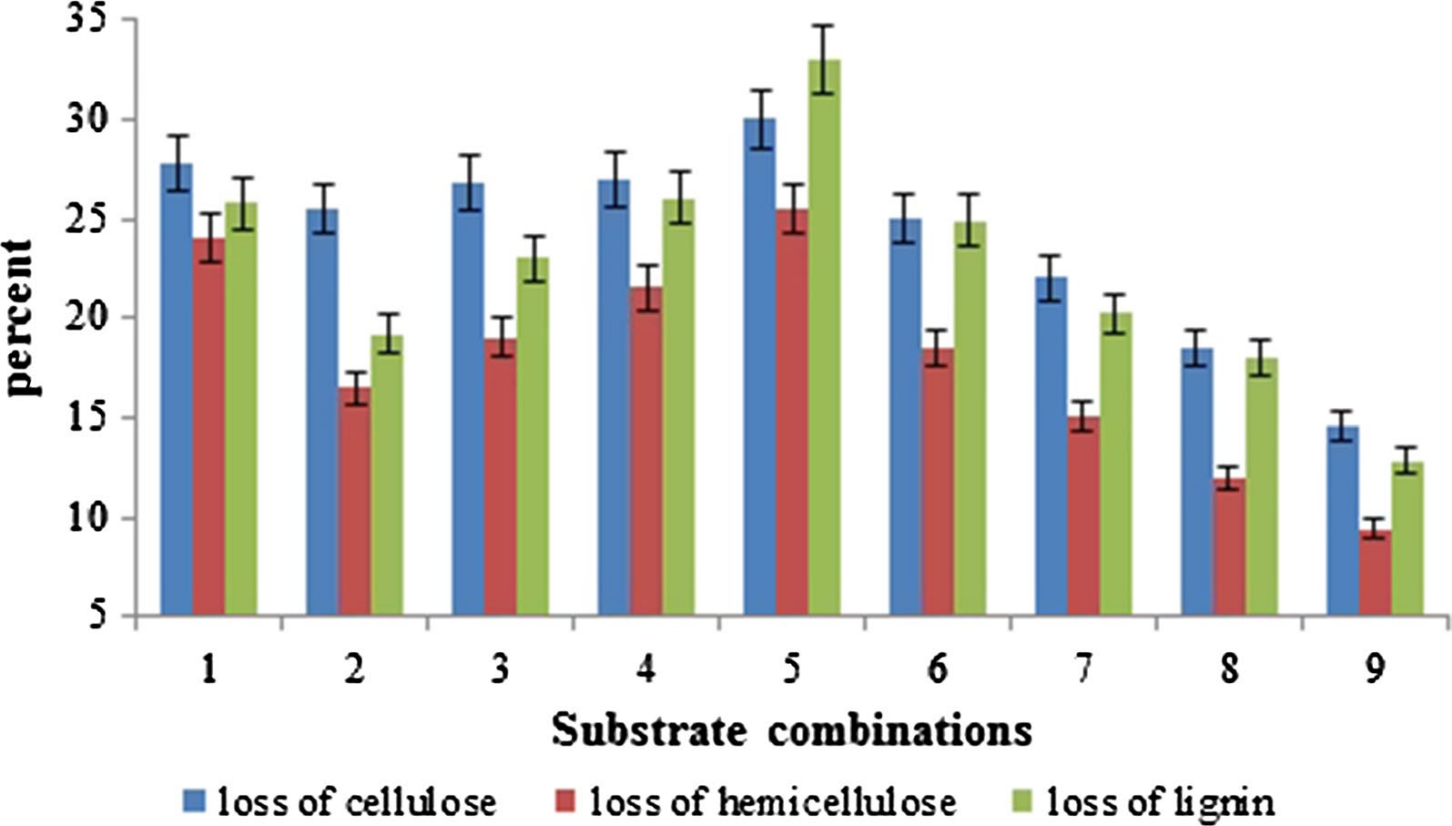
Degradation of cellulose, hemicellulose and lignin in uninoculated and inoculated (before and after fruiting) substrates

Degradation of cellulose, hemicellulose and lignin in uninoculated and inoculated (before and after fruiting) substrates is shown in Table 3 and Fig. 1. Cellulose degradation varied from 18.63 to 35.6 g. The highest degradation amount was for 50% ramie stalk medium. Hemicellulose degradation ranged between 3.10 and 12.9 g. Lignin degradation varied widely among the tested substrates, ranging from 6.62 to 18.9 g. Similar to cellulose, most hemicellulose and lignin degradation was observed in 50% ramie stalk medium (Table 3). *F. velutipes* degraded 12.7–32.0% lignin, 14.4–30.2% cellulose and 9.3–25.7% hemicellulose during cultivation on the different substrates (Fig. 1). An increase in ramie stalk content in proportions ranging 10–50% increased lignocellulose degradation. In general, it appears that lignin is more easily utilized than cellulose and hemicelluloses by *F. velutipes* (Fig. 1).

**Table 3:** Lignin, cellulose and hemicellulose content in the different substrates incubated with *Flammulina velutipes*

Substrate number	Lignin			Cellulose			Hemicellulose		
	g/(300 g dry substrate)								
	Control	Before fruiting	After fruiting	Control	Before fruiting	After fruiting	Control	Before fruiting	After fruiting
1	64.28 ± 5.53	63.02 ± 0.95	51.78 ± 5.24	103.72 ± 3.12	102.74 ± 4.26	77.22 ± 2.46	64.23 ± 6.87	63.78 ± 1.85	53.73 ± 1.07
2	62.54 ± 7.26	60.97 ± 1.26	47.94 ± 3.23	108.47 ± 2.41	106.23 ± 7.46	80.03 ± 6.57	59.52 ± 3.56	58.07 ± 4.68	48.22 ± 0.78
3	60.86 ± 3.45	58.74 ± 3.23	44.66 ± 2.61	113.21 ± 2.21	111.74 ± 9.46	82.01 ± 11.23	54.83 ± 4.44	54.02 ± 3.54	42.93 ± 2.12
4	59.15 ± 1.54	57.22 ± 2.42	40.25 ± 2.16	117.86 ± 1.98	115.66 ± 7.48	82.26 ± 6.47	50.14 ± 3.87	48.93 ± 5.67	37.24 ± 5.23
5	57.45 ± 2.68	55.13 ± 3.21	43.25 ± 2.79	122.48 ± 1.73	121.72 ± 9.87	91.98 ± 8.24	46.42 ± 2.99	45.72 ± 4.12	37.82 ± 2.23
6	55.73 ± 4.45	54.02 ± 4.23	44.43 ± 3.42	127.31 ± 2.24	126.54 ± 6.34	99.01 ± 7.26	40.72 ± 5.41	39.91 ± 5.62	34.52 ± 1.54
7	54.32 ± 5.44	53.15 ± 2.32	44.52 ± 2.98	132.14 ± 2.32	131.57 ± 4.45	107.64 ± 6.54	36.35 ± 6.17	35.93 ± 1.27	32.05 ± 2.14
8	66.14 ± 2.23	63.57 ± 2.78	49.04 ± 4.65	99.12 ± 1.65	98.76 ± 6.37	71.32 ± 5.66	69.23 ± 3.47	68.05 ± 5.35	52.63 ± 3.12
9	52.23 ± 1.53	50.12 ± 2.43	45.61 ± 3.32	128.98 ± 1.47	127.12 ± 7.24	110.35 ± 4.29	33.42 ± 5.61	32.18 ± 1.09	30.32 ± 0.98

### Cellulase activities

CMCase, 1,4-β-exoglucanase and 1,4-β-glucosidase activities in inoculated (before and after fruiting) substrates is shown in Table 4. CMCase activities varied from 3.02 to 12.63 U/mL. The highest CMCase activity was appeared in 50% ramie stalk medium after *F. velutipes* mushroom fruiting. Enzyme activities increased in most of the substrates after fruiting. Similar to CMCase, an increase in ramie stalk content, between 10 and 50%, result in a linear increase in 1,4-β-exoglucanase and 1,4-β-glucosidase enzymatic activities (Table 4). 50% ramie stalk medium had the highest 1,4-β-exoglucanase and 1,4-β-glucosidase activities (14.52 and 19.72 U/mL, respectively), whereas 98% ramie stalk medium had the lowest levels (4.65 and 5.76 U/mL, respectively). Similar to CMCase, after mushroom fruiting, enzyme activity increased before fruiting.

**Table 4:** Cellulolytic enzymes activities of *Flammulina velutipes* before and after fruiting

Substrate number	CMCase (U/mL)					
	1,4-β-Exoglucanase (U/mL)			1,4-β-Glucosidase (U/mL)		
	Before fruiting	After fruiting	Before fruiting	After fruiting	Before fruiting	After fruiting
1	4.53 ± 0.07	8.09 ± 0.07	6.23 ± 0.05	9.35 ± 0.07	9.25 ± 0.08	15.36 ± 0.08
2	4.89 ± 0.05	8.36 ± 0.05	6.78 ± 0.08	10.56 ± 0.08	9.93 ± 0.07	15.98 ± 0.06
3	5.46 ± 0.11	10.18 ± 0.09	7.25 ± 0.12	11.72 ± 0.06	10.87 ± 0.06	16.21 ± 0.05
4	6.58 ± 0.02	11.54 ± 0.07	8.59 ± 0.09	11.86 ± 0.07	11.28 ± 0.05	17.85 ± 0.02
5	7.92 ± 0.08^a^	12.63 ± 0.09^a^	9.98 ± 0.07^a^	14.52 ± 0.05^a^	13.85 ± 0.06^a^	19.72 ± 0.03^a^
6	5.53 ± 0.07	10.52 ± 0.05	7.29 ± 0.08	11.96 ± 0.12	12.12 ± 0.01	17.24 ± 0.08
7	4.49 ± 0.08	8.75 ± 0.07	5.14 ± 0.11	9.66 ± 0.07	10.68 ± 0.07	17.87 ± 0.09
8	7.23 ± 0.07	11.89 ± 0.05	9.62 ± 0.06	13.53 ± 0.09	12.26 ± 0.08	18.59 ± 0.04
9	3.02 ± 0.12	6.18 ± 0.08	4.65 ± 0.07	7.24 ± 0.12	5.76 ± 0.14	10.23 ± 0.08

^a^Significant at 0.05 level

### Hemicellulase activities

1,4-β-Xylosidase and xylanase activities in inoculated (before and after fruiting) substrates is shown in Table 5. 1,4-β-xylosidase activities varied from 3.17 to 9.88 U/mL. The highest 1,4-β-xylosidase activity was appeared in 50% ramie stalk medium after *F. velutipes* mushroom fruiting. Enzyme activities increased in most of the substrates after fruiting. Similar to 1,4-β-xylosidase, after mushroom fruiting, xylanase activity increased after fruiting.

**Table 5:** Hemicellulolytic enzymes activities of *Flammulina velutipes* before and after fruiting

Substrate number	Xylanase (U/mL)		1,4-β-Xylosidase (U/mL)	
	Before fruiting	After fruiting	Before fruiting	After fruiting
1	2.35 ± 0.04	3.95 ± 0.03	3.35 ± 0.13	6.08 ± 0.05
2	2.99 ± 0.03	4.29 ± 0.02	4.86 ± 0.05	7.14 ± 0.13
3	3.75 ± 0.09	5.25 ± 0.07	4.23 ± 0.07	7.58 ± 0.17
4	4.83 ± 0.11	6.57 ± 0.08	4.75 ± 0.11	8.37 ± 0.08
5	5.52 ± 0.02^a^	8.22 ± 0.09^a^	6.72 ± 0.08^a^	9.88 ± 0.09^a^
6	3.07 ± 0.08	5.45 ± 0.11	6.14 ± 0.05	8.34 ± 0.02
7	2.48 ± 0.07	3.98 ± 0.13	3.72 ± 0.08	7.65 ± 0.05
8	4.84 ± 0.06	6.99 ± 0.07	5.35 ± 0.05	8.57 ± 0.07
9	3.05 ± 0.03	3.24 ± 0.04	3.17 ± 0.06	4.89 ± 0.08

^a^Significant at 0.05 level

### Ligninolytic enzyme activity

Laccase and peroxidase activities in inoculated (before and after fruiting) substrates is shown in Table 6. Laccase and peroxidase activities were detected in all substrates were higher before fruiting than after fruiting. Laccase activities varied from 8.23 to 25.98 U/mL. The highest laccase activity was appeared in 50% ramie stalk medium before *F. velutipes* mushroom fruiting. Peroxidase activities varied from 3.78 to 24.84 U/mL. Peroxidase activities were affected significantly by increasing amount of ramie byproducts in proportions ranging 10–50%. It is obvious that laccase and peroxidase enzymes activities were associated with lignin degradation.

**Table 6:** Ligninolytic enzymes activities of *Flammulina velutipes* before and after fruiting

Substrate number	Laccase (U/mL)		Peroxidase (U/mL)	
	Before fruiting	After fruiting	Before fruiting	After fruiting
1	15.24 ± 0.11	9.37 ± 0.09	9.42 ± 0.06	7.09 ± 0.07
2	16.35 ± 0.16	10.45 ± 0.02	10.72 ± 0.09	7.23 ± 0.05
3	18.12 ± 0.09	12.06 ± 0.07	15.35 ± 0.12	9.08 ± 0.07
4	23.83 ± 0.03	13.15 ± 0.12	20.49 ± 0.07	14.65 ± 0.02
5	25.98 ± 0.08^a^	14.98 ± 0.07^a^	24.84 ± 0.11^a^	15.65 ± 0.12^a^
6	19.55 ± 0.13	10.25 ± 0.15	17.37 ± 0.05	11.58 ± 0.08
7	15.32 ± 0.07	9.15 ± 0.09	14.25 ± 0.03	10.37 ± 0.06
8	24.21 ± 0.06	14.04 ± 0.07	20.09 ± 0.05	15.06 ± 0.07
9	14.19 ± 0.17	8.23 ± 0.15	6.05 ± 0.13	3.78 ± 0.03

^a^Significant at 0.05 level

### Correlation coefficients (R) between biological efficiency and lignocellulose degradation, enzyme activities

After statistical analysis of correlation coefficients (R) between biological efficiency and lignocellulose degradation, enzyme activities, the results in Table 7 showed no significant correlation between increasing biological efficiency of *F. velutipes* and lignin, cellulose and hemicellulose degradation, but correlation between biological efficiency and activities of cellulase, hemicellulase and ligninolytic enzyme was positive.

**Table 7:** Correlation coefficients between biological efficiency and lignocellulose degradation, enzyme activities

Parameters	Biological efficiency (%)
Lignin content—control	0.528
Lignin content—before fruiting	0.525
Lignin content—after fruiting	−0.067
Cellulose content—control	−0.366
Cellulose content—before fruiting	−0.345
Cellulose content—after fruiting	−0.386
Hemicellulose content—control	0.495
Hemicellulose content—before fruiting	0.504
Hemicellulose content—after fruiting	0.362
CMCase activity—before fruiting	0.812^b^
CMCase activity—after fruiting	0.834^b^
1,4- β-Exoglucanase activity—before fruiting	0.731^a^
1,4- β-Exoglucanase activity—after fruiting	0.854^b^
1,4-β-Glucosidase activity—before fruiting	0.930^b^
1,4-β-Glucosidase activity—after fruiting	0.929^b^
Xylanase activity—before fruiting	0.63
Xylanase activity—after fruiting	0.710^a^
1,4-β-Xylosidase activity—before fruiting	0.800^b^
1,4-β-Xylosidase activity—after fruiting	0.855^b^
Laccase activity—before fruiting	0.814^b^
Laccase activity—after fruiting	0.827^b^
Peroxidase activity—before fruiting	0.710^a^
Peroxidase activity—after fruiting	0.719^a^

^a^Significant at 0.05 level

^b^Significant at 0.01 level

## Discussion

*F. velutipes* is one of the six most popular cultivated edible mushrooms in the world (Senik et al. 2015; Liu et al. 2017). In recent years, its consumption has increased and over 300,000 tons of *F. velutipes* are produced per year (Shi et al. 2017). Selecting an economic and efficient substrate material to reduce production costs has been an important consideration in *F. velutipes* cultivation. Ramie stalk is an agricultural residue and is generally disposed of by burning or burying which represents a major cause for environmental pollution. If it could be re-used wholly or partially as *F. velutipes* cultivation substrate, as a substitute for cottonseed hull or sawdust, the cost of cultivating mushroom should be reduced. In the present research, the possibility of using ramie stalk as a substrate for *F. velutipes* cultivation was tested and a deeper understanding on the bioconversion of the substrate was also discussed.

The optium C/N ratio for *F. velutipes* was 30/1 (Shi et al. 2012). In this study, it was observed that C/N ratio of 50% ramie stalk substrate was closest to 30/1. The maximum BE of fruiting bodies at 50% ramie stalk medium was reached 119%, which was significantly higher than values reported by other authors (Tang et al. 2016). The results also showed that lignocelluloses degradation peak appeared in 50% ramie stalk substrate. So variation in the C/N ratio in the cultivation medium affected the rate of lignocelluloses degradation and biological efficiency of *F. velutipes*. Results presented in this research indicated that C/N ratio of 98% ramie substrate group was lower than other tested substrates, suggesting that ramie stalks were only used as a supplement of wheat straw and cotton seed hull based substrates in *F. velutipes* cultivation.

*F. velutipes* is also known to degrade lignocelluloses by producing several extracellular secreted enzymes. The extracellular enzymes involve an ensemble of both oxidative enzymes and hydrolytic enzymes (Wang et al. 2015; An et al. 2016). Cellulose and hemicellulose are degraded by hydrolytic enzymes whereas lignin is degraded by oxidative enzymes (Doddapaneni et al. 2013; Zhuang et al. 2012). The *F. velutipes* genome and NGS-based RNA-Seq revealed a vast array of genes associated with lignin and carbohydrate degradation common to white rot fungi (Park et al. 2014). Various authors have tried to establish correlations between lignocellulose degradation and lignocellulolytic enzymes synthesis, biological efficiency, and lignocellulose degradation (Montoya et al. 2012). It is found that activities of endoglucanase, laccase and polyphenol oxidase were found to be more crucial for *Volvariella volvacea* yield on pasteurized substrate, while xylanase and β-glucosidase were more important for composted substrate (Ahlawat et al. 2008). In the present research, *F. velutipes* exhibited a higher cellulose and ligninolytic enzyme activity compared with hemicellulase enzyme activity with almost all substrates tested here. The positive relationship obtained in the present study between mushroom yield and activities of cellulase, hemicellulase and ligninolytic enzyme revealed that these enzymes are an important factor for fruit body formation. In conclusion, the biodegradation of ramie stalk by *F. velutipes* was evaluated by mushroom production and substrate utilization. This is the first report that compares the effect of ramie stalk supplementation of wheat bran and cornstarch, with or without cotton seedhulls on lignocellulolytic enzyme production, substrate degradation, and mushroom production in *F. velutipes*. The results of this study demonstrate that ramie stalk can be used as an effective supplement for increasing mushroom yield in *F. velutipes* and can increase the utilization efficiency of ramie stalks.

## Abbreviations

ANOVA: one-way analysis of variance
BE: biological efficiency

## References

[CR1] Adlakha N, Sawant S, Anil A, Lali A, Yazdani SS (2012). Specific fusion of beta-1,4-endoglucanase and beta-1,4-glucosidase enhances cellulolytic activity and helps in channeling of intermediates. Appl Environ Microbiol.

[CR2] Ahlawat OP, Gupta P, Dhar BL, Sagar TG, Rajendranath R, Rathnam K (2008). Profile of the extracellular lignocellulolytic enzymes activities as a tool to select the promising strains of *Volvariella volvacea* (Bull. ex Fr.) sing. Indian J Microbiol.

[CR3] An Q, Han ML, Wu XJ, Si J, Cui BK, Dai YC, Wu B (2016). Laccase production among medicinal mushrooms from the Genus *Flammulina* (*Agaricomycetes*) under different treatments in submerged fermentation. Int J Med Mushrooms.

[CR4] Aracri E, Roncero MB, Vidal T (2011). Studying the effects of laccase-catalysed grafting of ferulic acid on sisal pulp fibers. Bioresour Technol.

[CR5] Chen P, Yong Y, Gu Y, Wang Z, Zhang S, Lu L (2015). Comparison of antioxidant and antiproliferation activities of polysaccharides from eight species of medicinal mushrooms. Int J Med Mushrooms.

[CR6] Coconi-Linares N, Magana-Ortiz D, Guzman-Ortiz DA, Fernandez F, Loske AM, Gomez-Lim MA (2014). High-yield production of manganese peroxidase, lignin peroxidase, and versatile peroxidase in *Phanerochaete chrysosporium*. Appl Microbiol Biotechnol.

[CR7] Doddapaneni H, Subramanian V, Fu B, Cullen D (2013). A comparative genomic analysis of the oxidative enzymes potentially involved in lignin degradation by *Agaricus bisporus*. Fungal Genet Biol.

[CR8] Garcia-Maraver A, Salvachua D, Martinez MJ, Diaz LF, Zamorano M (2013). Analysis of the relation between the cellulose, hemicellulose and lignin content and the thermal behavior of residual biomass from olive trees. Waste Manag.

[CR9] Gomaa EZ (2013). Optimization and characterization of alkaline protease and carboxymethyl-cellulase produced by *Bacillus pumillus* grown on *Ficus nitida* wastes. Braz J Microbiol.

[CR10] Gupta S, Rohatgi A, Ayers CR, Patel PC, Matulevicius SA, Peshock RM, Markham DW, de Lemos JA, Berry JD, Drazner MH (2011). Risk scores versus natriuretic peptides for identifying prevalent stage B heart failure. Am Heart J.

[CR11] Huang Q, Jia Y, Wan Y, Li H, Jiang R (2015). Market survey and risk assessment for trace metals in edible fungi and the substrate role in accumulation of heavy metals. J Food Sci.

[CR12] Isikhuemhen OS, Mikiashvili NA, Adenipekun CO, Ohimain EI, Shahbazi G (2012). The tropical white rot fungus, *Lentinus squarrosulus* Mont.: lignocellulolytic enzymes activities and sugar release from cornstalks under solid state fermentation. World J Microbiol Biotechnol.

[CR13] Jing P, Zhao SJ, Lu MM, Cai Z, Pang J, Song LH (2014). Multiple-fingerprint analysis for investigating quality control of *Flammulina velutipes* fruiting body polysaccharides. J Agric Food Chem.

[CR14] Kang LZ, Zeng XL, Ye ZW, Lin JF, Guo LQ (2014). Compositional analysis of the fruiting body of transgenic *Flammulina velutipes* producing resveratrol. Food Chem.

[CR15] Levin L, Diorio L, Grassi E, Forchiassin F (2012). Grape stalks as substrate for white rot fungi, lignocellulolytic enzyme production and dye decolorization. Rev Argent Microbiol.

[CR16] Liu JY, Chang MC, Meng JL, Feng CP, Zhao H, Zhang ML (2017). Comparative proteome reveals metabolic changes during the fruiting process in *Flammulina velutipes*. J Agric Food Chem.

[CR17] Montoya S, Orrego CE, Levin L (2012). Growth, fruiting and lignocellulolytic enzyme production by the edible mushroom *Grifola frondosa* (maitake). World J Microbiol Biotechnol.

[CR18] Nakatani Y, Lamont IL, Cutfield JF (2010). Discovery and characterization of a distinctive exo-1,3/1,4-{beta}-glucanase from the marine bacterium *Pseudoalteromonas* sp. strain BB1. Appl Environ Microbiol.

[CR19] Park YJ, Baek JH, Lee S, Kim C, Rhee H, Kim H, Seo JS, Park HR, Yoon DE, Nam JY, Kim HI, Kim JG, Yoon H, Kang HW, Cho JY, Song ES, Sung GH, Yoo YB, Lee CS, Lee BM, Kong WS (2014). Whole genome and global gene expression analyses of the model mushroom *Flammulina velutipes* reveal a high capacity for lignocellulose degradation. PLoS ONE.

[CR20] Pasangulapati V, Ramachandriya KD, Kumar A, Wilkins MR, Jones CL, Huhnke RL (2012). Effects of cellulose, hemicellulose and lignin on thermochemical conversion characteristics of the selected biomass. Bioresour Technol.

[CR21] Senik SV, Maloshenok LG, Kotlova ER, Shavarda AL, Moiseenko KV, Bruskin SA, Koroleva OV, Psurtseva NV (2015). Diacylglyceryltrimethylhomoserine content and gene expression changes triggered by phosphate deprivation in the mycelium of the basidiomycete *Flammulina velutipes*. Phytochemistry.

[CR22] Shi M, Yang Y, Guan D, Zhang Y, Zhang Z (2012). Bioactivity of the crude polysaccharides from fermented soybean curd residue by *Flammulina velutipes*. Carbohydr Polym.

[CR23] Shi L, Chen D, Xu C, Ren A, Yu H, Zhao M (2017). Highly-efficient liposome-mediated transformation system for the basidiomycetous fungus *Flammulina velutipes*. J General Appl Microbiol.

[CR24] Studer MH, Demartini JD, Davis MF, Sykes RW, Davison B, Keller M, Tuskan GA, Wyman CE (2011). Lignin content in natural *Populus variants* affects sugar release. Proc Natl Acad Sci USA.

[CR25] Tang C, Hoo PC, Tan LT, Pusparajah P, Khan TM, Lee LH, Goh BH, Chan KG (2016). Golden needle mushroom: a culinary medicine with evidenced-based biological activities and health promoting properties. Front Pharmacol.

[CR26] Tsai SY, Huang EW, Lin CP (2017). Compositional differences of the winter culinary-medicinal mushroom, *Flammulina velutipes* (*Agaricomycetes*), under three types of light conditions. Int J Med Mushrooms.

[CR27] Varnai A, Makela MR, Djajadi DT, Rahikainen J, Hatakka A, Viikari L (2014). Carbohydrate-binding modules of fungal cellulases: occurrence in nature, function, and relevance in industrial biomass conversion. Adv Appl Microbiol.

[CR28] Vetrovsky T, Baldrian P, Gabriel J (2013). Extracellular enzymes of the white-rot fungus *Fomes fomentarius* and purification of 1,4-beta-glucosidase. Appl Biochem Biotechnol.

[CR29] Wang W, Yuan T, Cui B, Dai Y (2013). Investigating lignin and hemicellulose in white rot fungus-pretreated wood that affect enzymatic hydrolysis. Bioresour Technol.

[CR30] Wang W, Liu F, Jiang Y, Wu G, Guo L, Chen R, Chen B, Lu Y, Dai Y, Xie B (2015). The multigene family of fungal laccases and their expression in the white rot basidiomycete *Flammulina velutipes*. Gene.

[CR31] Wu M, Luo X, Xu X, Wei W, Yu M, Jiang N, Ye L, Yang Z, Fei X (2014). Antioxidant and immunomodulatory activities of a polysaccharide from *Flammulina velutipes*. J Tradit Chin Med.

[CR32] Xia Z (2015). Preparation of the oligosaccharides derived from *Flammulina velutipes* and their antioxidant activities. Carbohydr Polym.

[CR33] Yan ZF, Liu NX, Mao XX, Li Y, Li CT (2014). Activation effects of polysaccharides of *Flammulina velutipes* mycorrhizae on the Tlymphocyte immune function. J Immunol Res.

[CR34] Zhou C, Ye J, Xue Y, Ma Y (2015). Directed evolution and structural analysis of alkaline pectate lyase from alkaliphilic *Bacillus* sp. N16-5 for improvement of thermostability for efficient ramie degumming. Appl Environ Microbiol.

[CR35] Zhu S, Tang S, Tang Q, Liu T (2014). Genome-wide transcriptional changes of ramie (*Boehmeria nivea* L. *Gaud*) in response to root-lesion nematode infection. Gene.

[CR36] Zhuang X, Yu Q, Wang W, Qi W, Wang Q, Tan X, Yuan Z (2012). Decomposition behavior of hemicellulose and lignin in the step-change flow rate liquid hot water. Appl Biochem Biotechnol.

